# Harnessing electromagnetic fields to assist bone tissue engineering

**DOI:** 10.1186/s13287-022-03217-z

**Published:** 2023-01-11

**Authors:** Hongqi Zhao, Chaoxu Liu, Yang Liu, Qing Ding, Tianqi Wang, Hao Li, Hua Wu, Tian Ma

**Affiliations:** Department of Orthopedics, Tongji Hospital, Tongji Medical College, Huazhong University of Science and Technology, Wuhan, 430030 Hubei China

**Keywords:** Electromagnetic fields, Bone tissue engineering, Osteogenesis, Bone regeneration

## Abstract

Bone tissue engineering (BTE) emerged as one of the exceptional means for bone defects owing to it providing mechanical supports to guide bone tissue regeneration. Great advances have been made to facilitate the success of BTE in regenerating bone within defects. The use of externally applied fields has been regarded as an alternative strategy for BTE. Electromagnetic fields (EMFs), known as a simple and non-invasive therapy, can remotely provide electric and magnetic stimulation to cells and biomaterials, thus applying EMFs to assist BTE would be a promising strategy for bone regeneration. When combined with BTE, EMFs improve cell adhesion to the material surface by promoting protein adsorption. Additionally, EMFs have positive effects on mesenchymal stem cells and show capabilities of pro-angiogenesis and macrophage polarization manipulation. These advantages of EMFs indicate that it is perfectly suitable for representing the adjuvant treatment of BTE. We also summarize studies concerning combinations of EMFs and diverse biomaterial types. The strategy of combining EMFs and BTE receives encouraging outcomes and holds a promising future for effectively treating bone defects.

## Background

Bone is a robust organ that can regenerate completely under physiological conditions. However, large bone defects resulting from traumatic injuries [1], congenital defects [2], or tumours [3] are unable to form a callus and are commonly accompanied by high complication rates [4]. Usually, clinical interventions are required for the functional recovery of patients with large bone defects. Inert metallic bone fixation devices or autologous and allogeneic bone grafting are the gold standards for the current treatment of large bone defects [5]. However, they all possess potential risks such as pain [6], comorbidities associated with surgery [5], donor site morbidity [7], secondary surgery to remove the inert fixation [8], and the risk of disease transmission from the donor tissues [9]. To promote bone repair without causing the aforementioned risks, biomaterials functioned as bone substitutes have been substantially developed. Eventually, bone tissue engineering (BTE) emerged as a highly interdisciplinary research field [5]. The predominant role of bone tissue engineering materials (functioned as scaffolds) is dedicated to mimicking the biochemistry and structure of the natural bone extracellular matrix [10]. Thus, biomimetic scaffolds, which provide an appropriate three-dimensional environment and mechanical support for cells, can properly guide tissue regeneration [11]. Dong et al. [12] proposed that a typical strategy of BTE usually involves the following aspects: (1) construction of biomimetic scaffolds, (2) seeding of osteoprogenitor cells on scaffolds, (3) employment of exogenous pro-osteogenic factors, and (4) transplantation to bone defects sites. Mesenchymal stem cells (MSCs) are the most commonly used osteoprogenitor cells owing to their prominent capacity to proliferate and differentiate. Bone tissue engineering using MSCs has been proven to be an effective means for reconstructing rodent bone defects in many studies [13]. Besides, the successful clinical applications of MSCs-loaded BTE in enhancing bone formation within defects area have been firmly supported by a large amount of preclinical and clinical data [14]. However, there are still some obstacles and challenges in the extensive use of MSCs-loaded BTE in clinical situations such as high cost [15], comprised cell survival [3], and limited cell number for clinical cases with large defects [16]. And technological advances are required for maximizing cell, viability, vascular network formation, and osteogenic differentiation capacity [14]. Hence, alternative interventions and strategies are needed for assisting BTE, especially mesenchymal stem cells-loaded bone tissue engineering.

It is acknowledged that life on earth evolved in the company of a static magnetic field combined with a vertically oriented electrostatic field [17–19]. And many lifeforms, such as birds needing long-distance migration, can sense the magnetic field on earth for navigation [20]. Given the interaction between electric/magnetic fields and living organisms on earth, numerous efforts were paid to explore the biological effects induced by electromagnetic fields (EMFs). And an increasing body of studies confirmed the non-negligible impact of EMFs on various cell activities including cell proliferation, differentiation, cell cycle, apoptosis, DNA replication and expression, and cytokine expression [21]. With the progress that has been made in understanding the biological effects induced by EMFs, it has been exploited for a myriad of applications including helping bone fracture healing [22], osteoarthritis improving [23], pain-relieving [24], insulin sensitivity improving [25], intervertebral fusion [26], and wound healing [27]. Externally applied fields now emerge as promising tools for fixing complicated situations in tissue engineering applications owing to their potential in remotely manipulating the classic triad of cells, materials, and biochemical factors in engineering constructs [28]. Therefore, EMFs composed of electric fields and magnetic fields are candidate tools for the successful performance of BTE.

Stem cells are considered to be one of the basic elements in BTE [29]. Both electric fields and magnetic fields, which are components of EMFs, can stimulate the osteogenic differentiation of MSCs [30, 31]. By activating MSCs, EMFs show great potential for bone tissue engineering [21]. Additionally, mechanotransduction, the biological process of cells to sense, and respond to mechanical stimuli [32], can be affected by EMFs [33]. It is well-determined that mechanotransduction possesses a pivotal role in bone tissue homeostasis and BTE [34–36]. Therefore, mechanotransduction is also the connecting bridge between EMFs and BTE [33]. Conclusively, we assume that EMFs, which can be applied before and/or after implantation, are advantageous biophysical tools for BTE. This review aims to highlight the advantages associated with EMFs in assisting the performance of BTE and introduce the current studies on employing pulsed EMF (PEMF) and sinusoidal EMF (SEMF) to assist BTE in bone defects repair.

## Application procedures and characteristics of EMFs

**Fig. 1:**
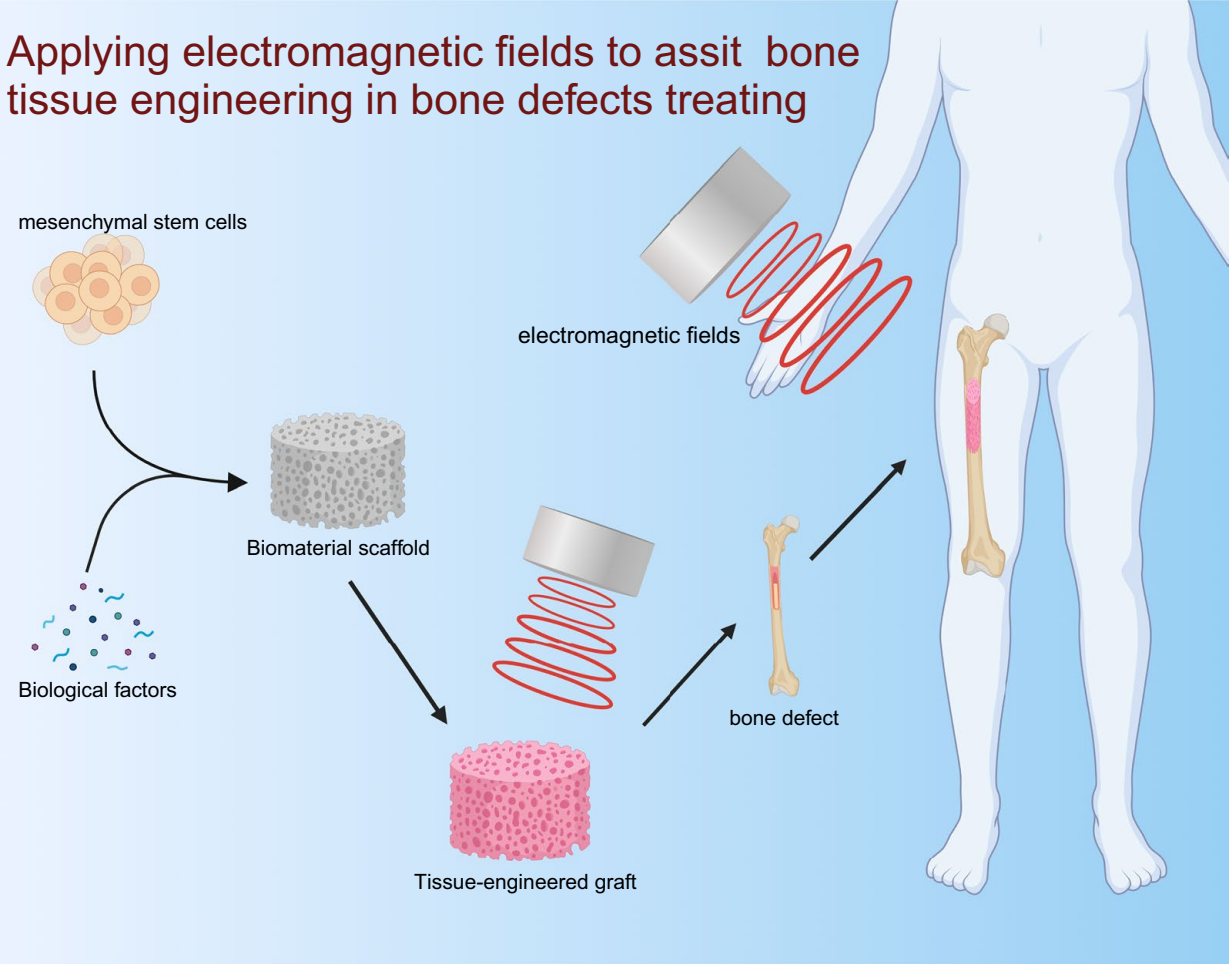
Harnessing electromagnetic fields to assist bone tissue engineering. Electromagnetic fields can be applied to stimulate loaded cell before and/or after implantation. This image was drawn by the authors. Created with BioRender.com

The ways in which EMFs’ stimulation was applied for BTE applications are summarized (Fig. 1). For BTE application, usually, MSCs that have been isolated and expanded would be seeded on tissue-engineered graft. Subsequently, the MSCs-laden constructs would be in vitro cultured with osteogenic medium under EMFs exposure for pre-osteogenic differentiation for a certain time. After the period of EMFs’ stimulation, MSCs-laden constructs will be implanted into the bone defects area. In addition, EMFs can also be applied after constructs implantation to sustainably promote bone regeneration in vivo.

**Fig. 2:**
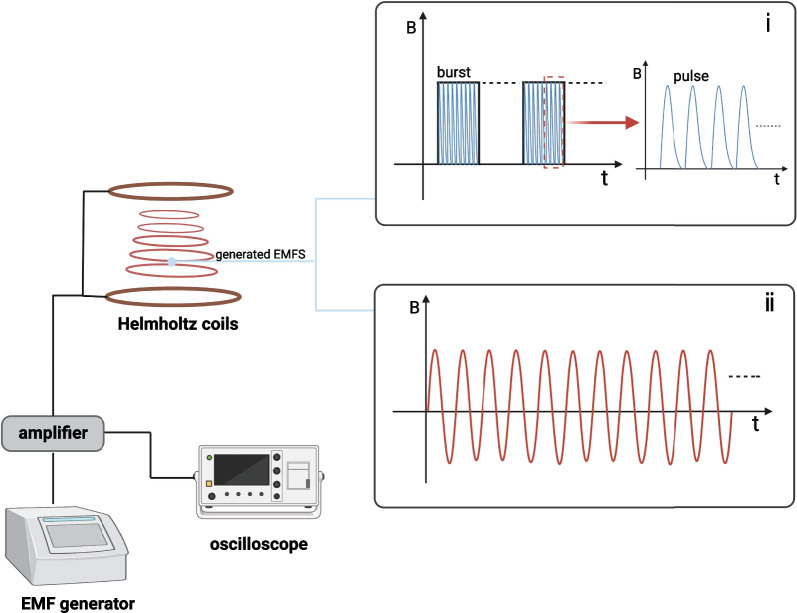
Scheme of electromagnetic fields generation system. An electromagnetic field generation system typically composed of a waveform generator, amplifier, oscilloscope, and Helmholtz coils. Two commonly used electromagnetic fields are presented: (1) pulsed electromagnetic field signal refers to the periodically repeated bursts composed of a certain amount of pulses [37]; (2) the non-pulsed sinusoidal electromagnetic field with continuous sinusoidal waveform. B refers to the magnetic flux density, *t* represents time. This image was drawn by the authors. Created with BioRender.com

Many kinds of EMFs currently exist, and PEMF and SEMF are two commonly used electromagnetic fields in treating bone defects. Both the two kinds of electromagnetic field show significant efficiency in helping bone regeneration. PEMF bursts are sent in an on-and-off manner, and the resulting PEMF signals refer to periodically repeated bursts which are composed of a certain amount of pulses [37]. Whilst the SEMF is non-pulsed, the sinusoidal magnetic waves are generated in a continuous manner during the exposure time [38]. Figure 2 shows the general equipment for generating electromagnetic field and the characteristics of the two electromagnetic fields.

## Biological effects of EMFs in bone tissue engineering application

Bone regeneration is an intricate, well-orchestrated process and involves multiple cell types and their interactions. EMFs can apply non-negligible impactions on various cell types involved in bone healing and are able to induce a broad range of cell activity. Consequently, EMFs can improve the performance of bone-tissue-engineered scaffolds through the following advantages.

### Cell adhesion

**Fig. 3:**
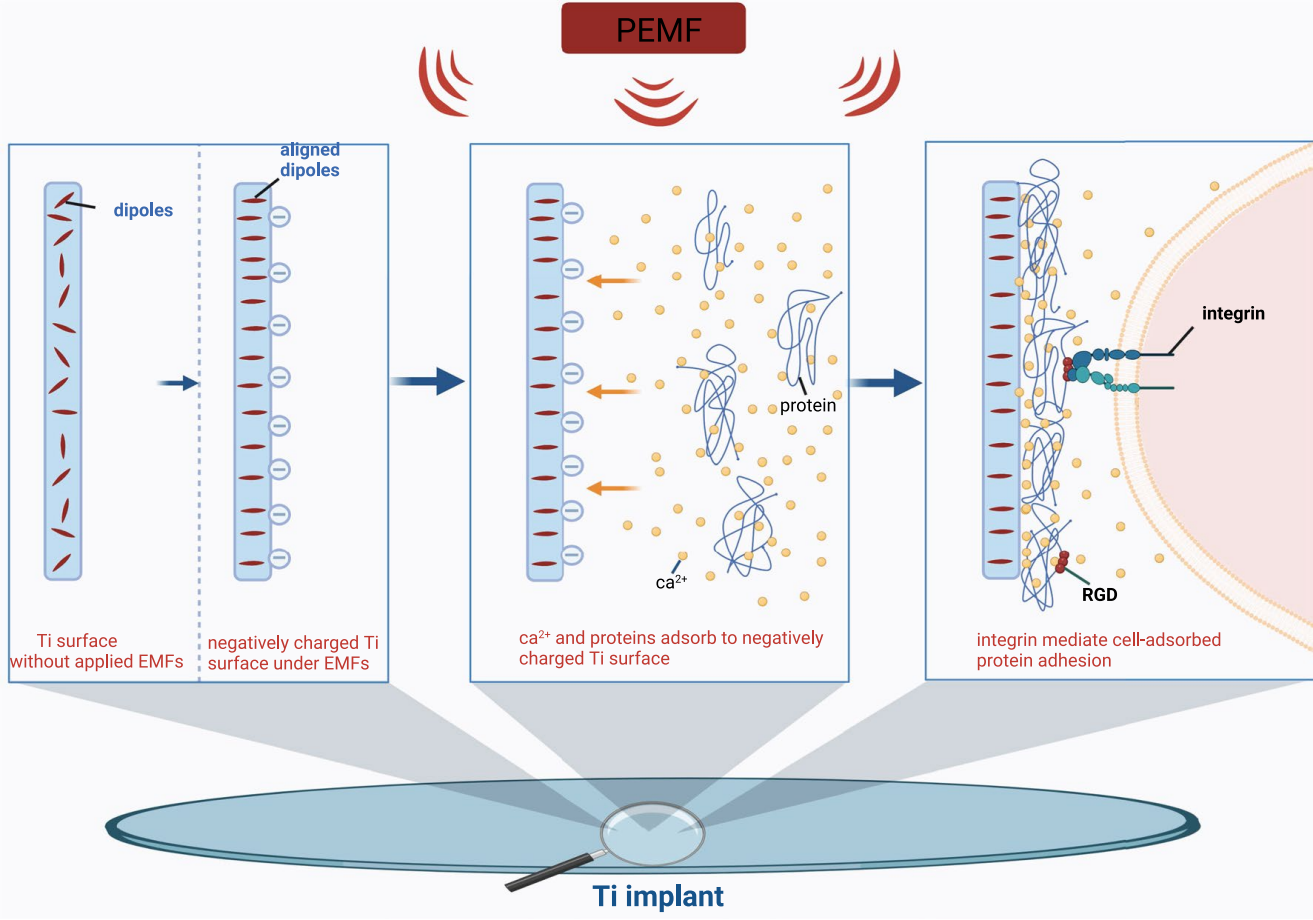
PEMF improves cell adhesion to Ti implant surface by promoting protein adsorption [42]. PEMF induces a negatively charged surface of Ti implant by making dipoles alignment. Cations mainly Ca^2+^ and proteins with positive charges adsorb onto negatively charged Ti surface. Cell adhesion-mediated motifs such as RGD embedded in adsorbed proteins mediate material-cell adhesion by binding to integrins located on cell membrane. *PEMF* pulsed electromagnetic field, *Ti* titanium, *RGD* Arg-Gly-Asp. This image was drawn by the authors. Created with BioRender.com

That stimulating cells to generate the expression of extracellular matrix for functional reconstruction of impaired tissues is a major concern of tissue engineering. The interactions between cells and engineered scaffolds, in which the biocompatibility of biomaterials is crucial, greatly affect the success of bone tissue engineering scaffolds [39]. Therefore, ensuring the biocompatibility of the engineered scaffolds is a critical prerequisite for their application. That good cell adhesion on the bone tissue engineering scaffolds’ surface is of great importance and is customarily taken as one of the important measurements for biocompatibility [40]. The positive effects of EMFs on cell adhesion have been determined by Chen et al. [41]. Their work showed that short-term exposure of EMFs (30 min/d) significantly promoted the cell adhesion and spreading of SCP-1 cell (an immortalized human mesenchymal stem cell), in addition, the short-term EMFs can even partly restore impaired SCP-1 cell adhesion caused by 5% cigarette smoke extract. Some studies further revealed that EMFs can certainly promote cell adhesion to the surface of tissue-engineered material by promoting protein adsorption [42–44]. Protein adsorption onto bone tissue engineering materials surface allows for cell adhesion and also possesses a vital role in determining the biocompatibility of materials [45, 46]. Wang et al. [42] reported that pulsed EMF (PEMF) actuation significantly increased protein adsorption to the titanium surface, which subsequently facilitates the initial adhesion of osteoblasts. And they assumed that PEMF stimulation induced a negatively charged surface of the titanium implant by making the dipoles aligned. Moreover, PEMF amplified the surface potential gradient of titanium implant [42]. Therefore, cations (mainly Ca^2+^) and proteins/peptides with positive charges would adhere to the negatively charged surface owing to the electrostatic interaction [47]. Subsequently, integrin on cell membrane, the main mediator in cell–matrix adhesion, recognizes and binds to cell adhesion-mediated motifs such as arginine-glycine-aspartic acid (RGD), which are embedded in adsorbed proteins [48, 49]. Additionally, a local higher Ca^2+^ concentration accelerates specific integrin receptor-mediated binding and contributes to focal adhesion formation [42, 50]. And the whole process is illustrated in Fig. 3. It is unknown whether SEMF could promote protein adsorption by such mechanism. Conclusively, using in vitro culture, it is determined that PEMF promotes cell adhesion to the material surface by promotion of protein adsorption, whereas it is still unclear whether PEMF is able to induce protein adsorption to scaffolds surface after implantation.

All cell types are capable of synthesizing and secreting matrix proteins to maintain the dynamic balance of extracellular matrix (ECM), which in turn dictates the cell fate and functions [51]. ECM is believed to mediate cell adhesion by binding to cell surface receptors (mainly integrin receptors), and ligands embedded in these ECM proteins play a significant role in that process [52]. For example, fibronectin and collagen, two principal components of ECM, are abundant in integrin receptor-binding domain and motif. Current studies showed that EMFs can directly stimulate expressions of ECM proteins such as fibronectin and collagen [53–55]. Additionally, a study carried out by Chen et al. [56] demonstrated the indirect effects of EMFs on ECM synthesis which were manifested by elevated expressions of collagen1, fibronectin, biglycan in SCP-1 cell cultured with conditioned medium from EMFs-exposed macrophages. These studies implied that EMFs might facilitate cell adhesion partly by promoting the expression of ECM proteins directly and indirectly. Lee et al. [57] demonstrated that EMFs significantly promoted human MSCs adhesion to the graphene substrate in vitro by stimulating expression levels of collagen type I and fibronectin. And their results of whole genome sequencing also confirmed that human MSCs stimulated by EMFs showed upregulated genes of ECM production [57]. Given that graphene is a two-dimensional crystal with unparalleled electric conductivity [58], the elevated ECM production might attribute to the electric activity between EMFs and graphene. In a recent study, it was proposed that reduced graphene oxide (RGO) under EMFs could generate magnetic moments, which subsequently evoke electric current [59]. It has long been identified that electric stimulation contributed to the increment of ECM-related protein production [60]. Therefore, the electric current induced by the combination of RGO and EMFs plays an essential role in elevated ECM production of human MSCs [59]. In conclusion, EMFs can intrinsically stimulate ECM production, which will be strengthened when EMFs combine with material with electrical responsiveness.

### Stem cells activities

**Fig. 4:**
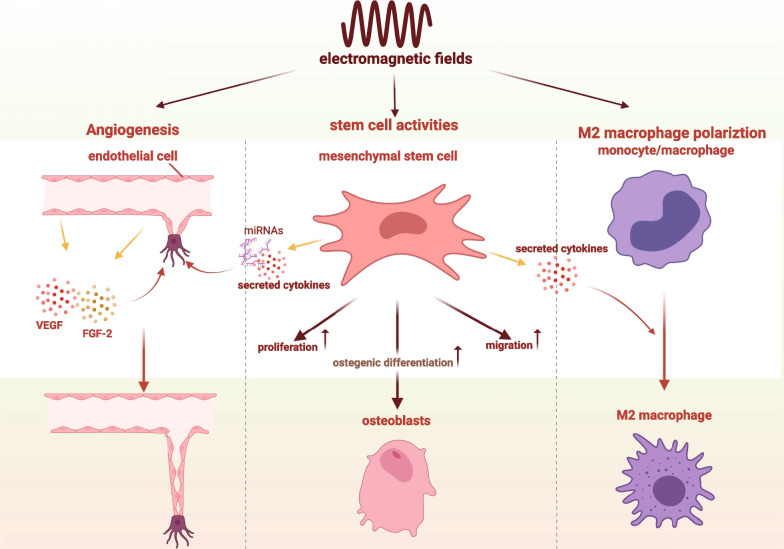
Advantages of electromagnetic fields in assisting bone tissue engineering. Advantages of electromagnetic fields including positive effects on stem cells, pro-angiogenesis, and preference of M2 macrophage polarization. This image was drawn by the authors. Created with BioRender.com

The unique capabilities of self-renewal and multilineage differentiation into cell lineages make stem cells the most suitable candidate for tissue engineering [61, 62]. Using the planarian regeneration model, Van Huizen et al. [63] demonstrated that weak magnetic force promoted stem cell proliferation and the subsequent differentiation by inducing changes in reactive oxygen species (ROS) accumulation and downstream heat shock protein 70 (Hsp70) expression. Similarly, it is confirmed that EMFs can induce non-negligible impacts on stem cell fate [64]. Plenty of studies carried out on MSCs from various sources suggested that EMFs’ stimulation positively affects the osteogenic differentiation and the expression of specific osteogenic markers (e.g. ALP, Runx2, osterix, and osteocalcin) [65]. Therefore, in most studies concerning the combination of EMFs and MSCs-loaded BTE, EMFs are employed to strengthen the osteogenic differentiation of stem cells (Fig. 4), whereas the clear mechanism of EMFs in osteogenic differentiation strengthening is complicated and has not been fully elucidated yet. One possible mechanism lies in ROS generation induced by EMFs. Recent studies believed that the involvement of EMFs in ROS production is a critical part of the cellular mechanisms underlying EMF-induced effects [66, 67]. Ehnert et al. [68] demonstrated that single EMF’s exposure stimulated ROS formation, and the induced ROS formation played an essential role in the improved osteogenic function of human osteoblasts by EMFs. Countless efforts were paid to explore and determine the pathways involved in stem cell osteogenic differentiation triggered by EMFs. Pathways implicated in pro-osteogenic differentiation of EMFs include Ca^2+^/CaM pathway, bone morphogenetic protein pathway (BMPs), tumour growth factor β (TGF-β) pathway, Wnt/β-catenin pathway, MAPK/ERK pathway, PI3K/Akt pathway [69, 70].

Aside from cell differentiation, cell proliferation and migration within the engineered scaffold is crucial for bone regeneration of critical size defects [71]. Using the human bone marrow-derived MSCs, our group confirmed that EMFs significantly promoted cell migration by activating intracellular Ca^2+^-dependent FAK/Rho GTPase migratory signalling [72]. Additionally, higher proliferation rate of MSCs resulted from EMFs’ stimulation is well-determined and has been widely demonstrated [73–75]. Therefore, after EMFs’ application, MSCs loaded on bone tissue engineering scaffolds would give rise to enhanced cell proliferation and migration, both of which are beneficial for bone regeneration. Previous studies showed that paracrine factors within conditioned medium of bone marrow-derived MSCs (BMSCs) could give rise to endothelial cell tube formation and macrophage recruitment in wound healing [76, 77], which implied the crosstalk between macrophage/endothelial cell and MSC take parts in tissue healing. And such crosstalk can be affected by EMFs [78]. Our group demonstrated that conditioned medium of BMSCs stimulated by EMFs showed prominent pro-angiogenetic capacity and osteoimmunomodulation effect [79]. And we further confirmed that it was the secreted cytokines within the conditioned medium that promote angiogenesis and M2 macrophage polarization (will be introduced later) [79]. A recent study similarly indicated that EMFs augmented the pro-angiogenetic capacity of MSCs by promoting the expression of miRNAs with intrinsic pro-angiogenic effect [80]. In conclusion, EMFs can be harnessed to impose beneficial effects on MSCs thereby extending and amplifying the beneficial role of MSCs in BTE.

### Angiogenesis

In light of the fact that bone is a complex, rigid, highly vascularized tissue, and skeletal vasculature plays a pivotal role in the process of bone regeneration, achieving proper blood supply is another essential element of bone tissue engineering [81]. A variety of strategies have been proposed to obtain better angiogenesis within scaffolds for effective bone regeneration [82–85]. Angiogenesis occurs when new capillaries develop from pre-existing vessels, in which process begins with growth factors (e.g. VEGF, FGF) binding to their homologous receptors on endothelial cells (ECs) followed by activation of these cells to produce relevant enzymes [86]. As early as 1988, EMF stimulation was reported to promote in vitro angiogenesis of endothelial cells [87]. It was further identified that EMFs promoted angiogenesis mainly by upregulating FGF2, and HIF-1α as well as its downstream growth factors, VEGF, in endothelial cells [88, 89]. Recently, a study carried out by Wang et al. [90] confirmed that EMFs counteract the bone loss in ovariectomy-induced osteoporosis mice by the expansion of CD31^hi^Emcn^hi^ endothelial cells. CD31^hi^Emcn^hi^ vessels (type H vessel) is a newly found vessel subtype in bone tissue, and it is characterized with the capability of coupling osteogenesis and angiogenesis [91, 92]. And studies suggested targeting type H vessel induction would be an effective strategy of osteoporosis prevention and BTE [83, 93]. Thus, it is possible that by EMFs’ application, the formation of type H vessel within bone tissue engineering scaffold could be augmented, which further facilitate bone regeneration. Conclusively, EMFs can induce the activation of diverse signalling pathways including the FGF and VEGF signalling pathways to enhance both osteogenesis and angiogenesis [70]. Given the pro-angiogenesis effect that EMFs possess (Fig. 4), it has been applied to facilitate angiogenesis in BTE [94, 95]. These results indicate that, by applying alone or combining with VEGF, EMFs indeed promote angiogenesis thus substantially promoting the overall performance of BTE in bone defect treating.

### M2 macrophage polarization

As a member of several cell lines that reside in bone marrow, monocytes/macrophages play a crucial role in bone homeostasis, repair, and anabolism, thus possessing the promising potential to aid bone regeneration [96]. At the early stage of inflammation, macrophages would swiftly migrate to the injured site in response to chemical cues after implant insertion [97]. Consequently, the response of macrophage to the engineered graft surface greatly determines the fate of the implant [98]. As an increasing body of evidence indicated the essential role of macrophages in bone regeneration, a broad range of strategies concerning macrophage manipulating were explored to facilitate better bone regeneration in BTE [99–102]. Moreover, the designing of engineered scaffolds favouring M2 macrophage polarization is one of the most effective ways. Stimulating by various signalling, macrophages characterized with diversity and plasticity may undergo M1 activation or M2 activation [103]. M2 macrophages are considered to be a pro-healing phenotype due to their ability to release high levels of the passivating cytokine such as IL-10 and TGF-β while generating low levels of inflammatory cytokines [104]. It has been reported that M2 macrophage polarization can be induced by magnetic force or electrical cues [105, 106]. Therefore, it is reasonable to consider that EMFs may modulate macrophage activity and induce macrophage polarization. Chen et al. [56] identified that EMFs with specific parameters significantly increased the protein levels of arginase I (a marker of M2 macrophage) in human peripheral blood mononuclear cells (hPBMC). Meanwhile, using the human Cytokine Array C5, they further found that the specific EMFs favoured a pro-healing secretome in hPBMC [56]. In another study carried out by Vinhas et al. [107], macrophages cultured on magnetic responsive materials can respond to PEMF and show an M2 phenotype. Their results showed that a marker associated with M2 macrophage (CD206) can only be detected in human macrophages stimulated by PEMF. And their another study also indicated that PEMF significantly promotes the expression of genes associated with M2 macrophage (Arg-1, MRC-1, Singlec-1) in THP-1 cells, a human monocytic cell line [108]. In addition, the study carried by Fu et al. [109] identified that microwave irradiation, a kind of electromagnetic field, combined with magnetic nanoparticles would conduct M2 macrophage polarization on RAW 264.7 cell line. In this study, macrophage membrane-enveloped Fe_3_O_4_/Au nanoparticles showed activation of macrophage to produce less inflammatory cytokines due to its property of electromagnetic field under microwave irradiation. In general, EMFs possess the potential to dictate M2 macrophage polarization (Fig. 4), which is one of the advantages of EMFs in improving the performance of BTE.

## EMFs’ applications in assisting bone tissue engineering

A wide range of material kinds and their combinations have been considered as potential options for bone tissue engineering applications. Koons et al. [5] summarized the material types for bone tissue engineering including natural polymers, synthetic polymers, bioceramics, and carbon-based nanomaterials. The combinations of EMFs and these material types as well as their combinations (composites) have been demonstrated by numerous studies.

### Common material types

EMFs have been concomitantly applied with diverse common material types including natural polymers (chitosan, keratin), synthetic polymers (poly(ε-caprolactone), poly(lactic-co-glycolic acid), poly(l-lactic acid)), calcium phosphates(hydroxyapatite, β-tricalcium phosphate), metal(titanium), and graphene (see Table 1).

**Table1:** Combinations of EMFs and common material types for bone regeneration

Materials	Types and parameters	Exposure time	Biological model	Results	
Keratin	PEMF 75 ± 2 Hz,2.0 ± 0.2 mT	1 h/day, 3 weeks	Osteoblast-like cells (SAOS-2)	PEMF boosted the osteogenic differentiation and bone matrix production of osteoblast-like cells on keratin scaffold	Bloise et al*.* [127] 2020
Chitosan	PEMF 75 Hz,18–30 Gauss	2 h/day, 3 weeks	Osteoblasts (7F2, VA, CRL-12557, ATCC)	EMF enhanced the proliferation and mineralization of osteoblasts on chitosan substrate	Lin et al. [124] 2010
PCL	PEMF 50 Hz,1.0 mT	6 h/day, up to 3 weeks	Human ADSCs	PEMF augmented osteogenic differentiation of ADSCs on PCL scaffold without biological factors	Arjmand et al. [112] 2018
PCL	PEMF 50 Hz,1.0 mT	6 h/day, up to 3 weeks	Human iPSCs	PEMF augmented osteogenic differentiation of iPSCs on PCL scaffold without biological factors	Ardeshirylajimi et al. [113] 2018
PLGA	PEMF 7.5 Hz,0.13/0.24/0.32 mT	Continuous exposure, up to 18 days	Osteoblasts derived from calvaria of rats	PEMF stimulation with specific parameters had an effect on regulating the osteoblast proliferation and differentiation	Tsai et al. [118] 2007
PLGA	PEMF 50 Hz,0.5 mT	8 h/day, up to 12 days	Cortical bone of mice femurs	EMF had a positive effect on enhancing early implant osseointegration in trabecular bone and a greater degree of bone mineralization and maturation	Zhong et al. [119] 2012
CaP	PEMF 7.5 Hz,4.8/8.7/12.2 μV/cm	2 h/day, up to 10 days	Mouse osteoclasts	PEMF with different intensities regulated osteoclastogenesis and bone resorption in a bone-biomimicking environment by modulating OPG, RANK ligand and M-CSF	Chang et al. [129] 2005
CaP	PEMF 15 Hz,16 Gauss	8 h/day, up to 24 days	Human MSCs	PEMF augmented the biological response of BMP-2 on MSC in a bone-biomimicking environment	Schwartz et al. [130] 2008
CaP	PEMF 15 Hz,16 Gauss	8 h/day, up to 12 days	Human MSCs and human osteoblast-like cells	Under PEMF exposure, osteoblast-like cells cultured on CaP has a higher OPG production than cells cultured on tissue culture polystyrene plastic	Schwartz et al. [131] 2009
HAp	PEMF 75 Hz,1.6 mT	6 h/day, 3 weeks	Rabbits femur	PEMFs accelerated HA osteointegration in trabecular bone	Fini et al. [136] 2002
HAp	CC-PEMF 16.0 Hz,10 V,7.8 Volt/m	Continuous exposure, 45 days	Hindlimb suspension rat model	A combination of PEMF and HAp nanoparticles has potential to counteract bone loss	Prakash et al. [137] 2009
β-TCP	PEMF 50 Hz,1.0 mT	2 h/day, up to 12 weeks	Rat ADSCs and skull defects model	A combination of the β-TCP scaffold and PEMF significantly promote repair of critical defect of rat skull	Liang et al. [140] 2019
Ti	PEMF 15 Hz, 9.6 Gauss	Continuous exposure, up to 45 days	Rat osteoblasts	PEMFs enhanced the osteoblast compatibility on different Ti surfaces (flat, micro, and nano), while the combination of PEMFs and macro-/nano-surface Ti had a better result	Wang et al. [42] 2014
Ti	PEMF 75 Hz,2.0 mT	10 min/day, up to 28 days	Human BMSCs	PEMF promoted osteogenic differentiation and ECM production of human BMSCs cultured on nano-surface Ti	Bloise et al. [154] 2018
Ti	PEMF 15 Hz, 2.0 mT	2 h/day, 8 weeks	Bone defect of alloxan-induced diabetic rabbit	PEMF improved bone architecture and porous Ti osseointegration by regulating bone anabolism	Cai et al. [152] 2018
Ti	PEMF 15 Hz, 2.0 mT	2 h/day, 4 weeks	Glucocorticoid-treated bone defect rabbit model	PEMF improved bone mass, strength and porous implant osseointegration in glucocorticoid-treated rabbits	Cai et al. [153] 2020
Graphene	PEMF 50 Hz,0.6 ± 0.05 mT	Continuous exposure	Human alveolar bone marrow stem cells	The combination of RGO and PEMFs enhanced proliferation, differentiation, and ECM production of human MSC	Lim et al. [59] 2016

*PCL* poly(ε-caprolactone), *PLGA* poly(lactic-co-glycolic acid), *HAp* hydroxyapatite, *β-TCP* β-tricalcium phosphate, *Ti* titanium, *CaP.* calcium phosphate, *PEMF* pulsed electromagnetic fields, *CC-PEMF* capacitive coupling of pulsed electromagnetic field, *ADSCs* adipose-derived mesenchymal stem cells, *iPSCs* induced pluripotent stem cell, *BMSCs* bone marrow/mesenchymal stem cells, *BMP-2* bone morphogenetic protein-2, *OPG* osteoprotegerin, *RANK* receptor activator of nuclear kappa-B, *M-CSF* macrophage-stimulating factor, *RGO* reduced graphene oxide

#### Synthetic polymers

Synthetic polymers can mimic the normal extracellular matrix, and possess a pivotal position in tissue engineering [110]. The significant role of poly(ε-caprolactone) (PCL) in tissue engineering has already been well determined because of its facility of processing into long-term degradable engineered graft and its authorized security approved by the US Food and Drug Administration (FDA) [111]. Biological factors are essential players in BTE due to their role of providing correct biological signalling to guide cell differentiation and tissue growth; however, their application also brings some concerns about potential side effects [112]. In most studies concerned with a combination of BMSCs-loaded BTE and EMFs, the osteogenic medium is required for osteogenic induction, whereas, in a study carried out by Ardeshirylajimi et al. [113], EMFs’ stimulation without osteogenic medium management promoted expressions of osteogenic-related gene markers in human induced pluripotent stem cells (iPSCs) seeded on PCL nanofibres. And surprisingly, for iPSCs cultured on PCL nanofibres, EMFs’ stimulation showed a similar effect as an osteogenic medium on ALP activity, calcium content as well as expression of osteogenic gene markers [113]. The similar pro-osteogenic effect of a combination of EMFs and PCL nanofibres was also confirmed within adipose-derived mesenchymal stem cells (ADSCs) [112]. Among adult stem cells (stem cells resided in practically all organs and tissues of the adult organism, e.g. BMSCs), ADSCs are believed to be the most advantageous for tissue engineering. And it is ascribed to its ease of harvesting and high abundance in corresponding derived source tissue in comparison to BMSCs [62]. Consequently, the combination of EMFs and ADSCs-load PCL nanofibres seems a promising strategy in BTE as EMFs could be a potential alternative option for osteogenic growth factors [112].

Poly(lactic-co-glycolic acid) (PLGA) is a copolymer of lactic acid and glycolic acid. Its degradation rate can be adjusted by changing the ratio of lactic acid to glycolic acid [114–116]. With tunable degradation rates, good mechanical properties, and processability, PLGA is regarded as a popular and biodegradable biomaterial in BTE [117]. It has been reported that EMFs exposure with specific parameters promoted proliferation and ALP expression of rodent osteoblasts/BMSCs seeded on PLGA scaffold, which indicated the potential of EMFs to aid PLGA applied-BTE [118, 119].

#### Natural polymers

Natural polymers possess some prominent features of higher biocompatibility, excellent biodegradability, and no toxicity, and they have been widely applied for BTE and receive encouraging results [120].

Chitosan, the partially deacetylated form of chitin, is capable of promoting tissue growth and proliferation [121]. Due to its novel properties such as biocompatibility, biodegradability, and wound-healing activity, chitosan holds a promising future in tissue engineering applications [122]. Furthermore, chitosan is suitable for fabricating biomimetic scaffolds because it has a similar structure to glycosaminoglycans, an essential structural component of bone matrix [123]. A study demonstrated that EMFs enhanced the proliferation and mineralization of osteoblasts seeded on chitosan scaffold, which indicated the applicability of a combination of EMFs and chitosan scaffold in large bone defects treatment [124].

Keratin, which is the main component of feathers, hair, hoofs, and wool, has some intrinsic characteristics to assist cell adhesion, proliferation, and tissue regeneration. With these inherent biological characteristics and excellent biocompatibility, keratin-based biomaterials are widely applied for many biomedical applications such as bone morphogenetic protein carriers, ocular surface reconstruction, wound healing, and nerve regeneration [125, 126]. Bloise et al. identified that wool keratin scaffold can be applied as osteoconductive biomaterials under the joint action of PEMF and osteogenic factors. The combination of PEMF and osteogenic factors rendered osteoblast-like cells with higher calcified bone matrix deposited on wool keratin scaffolds [127].

#### Calcium phosphates

Calcium phosphates can mimic carbonated hydroxyapatite-the inorganic composites of bones, which makes it the most common bioceramics in BTE. And a broad range of biomaterials featuring calcium phosphates such as hydroxyapatite and β-tricalcium phosphate has been demonstrated [5]. It is generally believed that the osteoinductive characteristic of calcium phosphates comes from its releasing of Ca^2+^ and PO_4_^3−^, both of which facilitate the bone bone-like apatite to form on the surface of calcium phosphates, and these two inorganics are critical factors in bone matrix mineralization [128]. Due to the prominent biomimicking property, calcium phosphates are commonly used as culture substrates to mimic a bone-like environment. And with the calcium phosphates substrates, the effects of EMFs on MSCs, osteoblast, and osteoclasts in a bone-like environment were elucidated [129–131]. Generally, EMFs promote the osteogenic differentiation while inhibiting bone resorption in a bone-like environment.

Hydroxyapatite (HAp) is a well-known and biocompatible bioceramic that suits for fabricating orthopaedic and dental implants [132]. However, HAp implants would be very slowly replaced by host bone after implantation; thus, the induction of bone growth into hydroxyapatite is difficult [133], whereas the success of osteointegration, namely the connection between the peri-implant bone and the implant surface, substantially influences the long-term usage of implants [134]. As EMFs are capable of non-invasively promoting osteogenesis, some studies demonstrated that better osteointegration of HAp implants and host bone could be achieved by applying EMFs’ stimulation [135, 136]. These studies identified the feasibility of employing EMFs to avoid implant clinical failure by improving the implant osteointegration. Compared to conventional hydroxyapatite implants, nanoscale HAp is likely a better choice for biomedical applications [133]. And the combination of HAp nanoparticles and EMFs seems to be an effective way to counteract bone loss. Prakash et al. [137] suspended rat tails for 45 days to mimic bone lose cause by microgravity and treated these hindlimb suspension (HLS) rats with different treatments (PEMF, HAp nanoparticles, PEMF + HAp nanoparticles) for another 45 days. They found that PEMF failed in restoring bone mineral density (BMD) in HLS rat femur (*P* < 0.5, compared with control) while HAp nanoparticles partially restored BMD in osteoporotic femur (*P* < 0.02, compared with control), whereas HLS rat treated by PEMF + HAp nanoparticles completely restored BMD and have a comparable BMD level (*P* < 0.01, compared with control) to rat without HLS management. The inadequate mechanical strength of HAp restricts its abroad application, and to overcome this drawback, HAp-reinforced nanomaterials obtained by doping with metals (magnetic particles) or other biomaterials have been demonstrated and received increasing attention [138]. A recent study by Fernandes Patrício et al. [139] indicated that magnetic microspheres obtained by doping HAp with iron were capable of carrying and releasing BMP-2 at a low dose for a certain time, and the release efficiency of BMP-2 could be elevated by EMFs’ stimulation. And they pointed out that such a combination of EMFs and magnetic HAp microspheres would be a new therapeutic strategy to meet clinical needs in bone cement and scaffolds for local bone replacements [139]. β-tricalcium phosphate(β-TCP) is an osteoinductive ceramic that can combine with EMFs to treat rat skull defects [140]. It is reported that iron-doped β-TCP significantly upregulates ALP expression and calcium deposition when an external PEMF was applied [141], which implies that a combination of EMFs and iron-doped bioceramics may be a new method for bone regeneration.

#### Titanium

Ti and its alloys are ideal biomaterials for bone-tissue-engineered implants [142, 143]. Implant loosening resulting from inadequate bone integration or the development of fibrous tissue is still one of the leading reasons for titanium implant failures in its clinical application [144]. As mentioned above, EMFs employment is efficient for promoting the osteointegration of hydroxyapatite implants and host bones. Thus, EMFs are similarly expected to prevent Ti implant loosening resulting from diverse conditions by improving osteointegration [145, 146]. For instance, osteolysis-induced looseness around the implants prosthesis is a common complication of orthopaedics [147]. Veronesi et al. [148] demonstrated that PEMF was a safe, and conservative treatment for counteracting periprosthetic osteolysis in rats. In their study, the histological and histomorphometric results demonstrated that bone-to-implant contact (BIC), a significant indicator of osteointegration, was significantly elevated (*P* < 0.0005, compared to osteolysis rats without treatments) after employing PEMF for 6 h/day for 60 days. And both fibrous capsule thickness (*P* < 0.005) and osteoclasts number (*P* < 0.0005) were dramatically decreased by PEMF [148]. The reduction in fibrous tissue formation around implants may ascribe to the induction of EMFs in the attenuation of mast cell infiltration around implants [149]. It is acknowledged that metabolic abnormalities detrimentally affect implant osteointegration and patients with diabetes mellitus are more associated with implant failure [150, 151]. Considering the increasing prevalence of diabetes mellitus, effective methods for improving osseointegration in diabetic patients are in urgent need. A study carried out by Cai et al. [152] indicated that EMFs management seemed a valuable method for combatting Ti implant loosening within type 1 diabetic rabbits. Their results confirmed that EMFs significantly improved the impaired bone architecture and mechanical properties incited by diabetic mellitus, which would subsequently help for the success of implant therapy in diabetic rabbits. Moreover, EMFs employment is also effective in improving glucocorticoid-impaired Ti implant osseointegration [153]. Conclusively, externally applied EMFs can effectively counteract Ti implant loosening resulting from diverse pathological conditions.

A vast variety of implant surface modification techniques such as topography modification has been developed to accelerate Ti implant osseointegration [144]. Wang et al. explored the effects of EMFs on the functions of osteoblasts on three types of titanium surfaces (flat, micro, and nano). They identified the positive impaction of EMFs on osteoblasts seeded on all three topographies including increasing cell adhesion, cell proliferation, extracellular matrix mineralization nodules, and expression of osteogenesis-related genes (BMP-2, OCN, Col-1, ALP, Runx2 and OSX) [42]. Bloise et al. similarly demonstrated that EMFs with clear pro-osteogenic effects can combine with Ti implants of nano-topography surface and might be an effective adjuvant treatment to improve the osseointegration of Ti implants [154].

### Composites

Composite materials have been considered a better choice for bone tissue engineering due to their ability to outperform their individual constituents [5]. For example, by adding poly(l-lactic acid) (PLLA), a member of synthetic polymers, into HAp, the resulting PLLA/HAp composite scaffold can not only overcome the disadvantages of HAp in unsatisfactory degradability and brittleness but also possesses higher cell viability in comparison to pure PLLA polymer scaffolds [155]. Hence, the studies on combining EMFs with composites including magnetic and non-magnetic composites are extensive (see Table 2).

**Table 2:** Combinations of EMFs and composites for bone regeneration

Composites	Parameters	Exposure time	Biological model	Results	
PLLA/HAp	SEMF 15 Hz, 1.0 mT	4 h/day, up to 12 weeks	Rat BMSCs and rat critical-sized calvarial defect model	SEMF promoted the proliferation and osteogenic differentiation of the BMSCs Cell-loaded constructs showed a higher new bone formation and vascularization after EMF stimulation	Tu et al. [95] 2020
PCL/HAp	SEMF 15 Hz, 0.3 mT	4 h/day, up to 8 weeks	Rat BMSCs and critical-sized calvarial defect model	SEMF promote the osteogenic potential of BMSCs and enhanced the paracrine function of BMSCs to facilitate bone regeneration	Li et al. [79] 2021
PCL/HAp	SEMF 15 Hz, 1.0 mT	4 h/day, up to 12 weeks	Rat BMSCs and subcritical cranial defect model	SEMF with VEGF enhanced the osteogenic differentiation and endothelial differentiation of rBMSCs	Chen et al. [94] 2019
HAp/collagen	SEMF 15 Hz, 1.0 mT	4 h/day, up to 14 days	Rabbit BMSCs and femur condyle defect model	EMF enhanced the osteogenic differentiation of BMSCs and promoted bone regeneration within scaffold	Wang et al. [162] 2019
CMC-grafted-PCL	EMF 50 Hz, 31.4 μT	1 h/day, up to 14 days	ADSCs	Grafted CMC showed osteoinductive effects on stem cells, which can be amplified by EMFs and β-carotene	Shapourzadeh et al. [157] 2020
PVA/PES	PEMF 50–400 Hz,30 mT	8 h/day, up to 14 days	ADSCs	The osteogenic potential of ADSCs seeded on the PVA/PES scaffold could be augmented by EMF	Heydari Asl et al. [163] 2018
PVDF/PANI	PEMF 50 Hz,1.0 mT	6 h/day, up to 14 days	Human DPSCs	PEMF significantly promoted protein adsorption on PVDF/PANI, and a combination of piezoelectric composite and electromagnetic stimulation has a better osteogenic induction	Mirzaei et al. [168] 2019
PVDF-coated PCL-TCP	PEMF 50 Hz,0.6 mT	30 min/day, up to 28 days	Murine MC3T3-E1 cells	Cells on PVDF-coated PCL-TCP showed well morphology and better osteogenic differentiation under PEMF exposure	Dong et al. [47] 2021
Magnetic TCP nanoparticles	PEMF 15 Hz, 16 V	4 h/day, up to 14 days	Human BMSCs	Intrinsically magnetic nanoparticles synergized with PEMF to stimulate osteogenic differentiation of BMSCs	Habib et al. [141] 2021
Magnetic PLLA /n-HAp	PEMF 100mT	Continuous exposure, up to 21 days	Rabbit BMSCs	Magnetic scaffold induced the osteogenic differentiation with the action of the PEMF	Huang et al. [177] 2017
Mg.ATP-decorated magnetic nanocomposites	EMF 0.9 T	30 s/3 day, 7 days	Mouse ADSCs	Mg.ATP-decorated magnetic nanocomposites showed augmented osteogenic differentiation and cell adhesion in the presence of EMF	Meshkini et al. [179] 2022
Magnetic microspheres	PEMF 75 ± 2 Hz, 2.0 ± 0.2 mT	Continuous exposure, up to 14 days	Human MSCs	The release of BMP-2 from magnetic microspheres can be accelerated due to the stimulation of PEMF	Fernandes et al. [139] 2021

*PCL* poly(ε-caprolactone), *HA* hydroxyapatite, *CMC* carboxymethyl chitosan, *PLLA* poly(l-lactic acid), *PVDF* polyvinylidene fluoride, *PANI* polyaniline, *PVA* polyvinyl alcohol, *PES* polyethersulfone, *EMF* electromagnetic fields, *SEMF* sinusoidal EMF, *PEMF* pulsed EMF, *BMSCs* bone marrow mesenchymal stem cells, *ADSCs* adipose-derived mesenchymal stem cells, *DPSCs* dental-pulp-derived stem cells

#### Non-magnetic composites

Non-magnetic composites here refer to conventional composites without magnetic properties. As mentioned above, PLLA/HA is superior to its individual constituents and thus represents a promising composite for BTE. Our group has demonstrated the feasibility of a combination of EMFs and PLLA/HAp scaffold in dealing with rat critical-sized calvarial defects [95]. Besides PLLA, PCL is another commonly used synthetic polymer to overcome the brittleness of HA owing to its high mechanical properties and approved safety. Our work showed that the combination of EMFs and PCL/HAp scaffold also obtained pleasing outcomes in treating rat bone defects [79, 94]. In the study carried out by Li et al., rat BMSCs-laden-PCL/HAp scaffolds were pre-treated with osteogenic medium and SEMF for 7 days (4 h/d) before in vivo implantation. And 8 weeks later, calvarial defect implanted with such scaffolds had more new bone area (*P* < 0.01) than rats received BMSCs-laden-PCL/HAp scaffolds pre-treated with osteogenic medium only [79]. Another example of composites composed of synthetic polymers and natural polymers is PCL/carboxymethyl chitosan (CMC). It has been reported that PCL/CMC scaffold fabricated by the electrospinning technique is suitable for BTE [156]. By grafting CMC onto PCL nanofibres, Shapourzadeh et al. revealed that grafted CMC endowed the composite scaffold with the ability to self-differentiate stem cells, and the osteoinductivity could be further augmented by EMFs employment or β-carotene management [157]. Collagen, the most abundant protein in the extracellular matrix, is the major structural element of all connective tissues [158]. Hence, collagen is believed to be an ideal biomaterial for tissue engineering related to skin, bone, and cartilage [159]. However, pure collagenous materials lack adequate mechanical strength and stiffness, and to counteract these flaws of collagen, inorganic materials are usually added to collagen for fabricating composite scaffolds [160]. And it is usually to disperse HAp into collagen to fabricate HA/collagen scaffold, a bioinspired composite suited for BTE application [161]. Our group previously demonstrated that EMFs employment was promising for improving the effectiveness of HA/collagen scaffold in rabbit femur defects repairment [162]. And the results indicated that BMSCs-laden-HA/collagen scaffold stimulated by SEMF successfully promote bone regeneration within the defect and bone integration between the graft and host bone in rabbit femur [162]. Other composites such as polyvinyl alcohol/polyethersulfone is also feasible for constructing tissue-engineered scaffolds and for combining with EMFs to stimulate osteogenesis [163].

Piezoelectricity refers to the ability to generate electric activity in response to mechanical stress and vice versa [164]. Piezoelectric materials have attracted much attention for providing electrical stimulation, a vital player in cell biological activity, to cells to promote tissue regeneration [165]. The natural bone has prominent piezoelectricity and it can physiologically generate electrical stimulation to enhance bone growth in response to mechanical stress, thus piezoelectric materials are suitable for fabricating bone tissue-engineered scaffolds [166]. Externally applied electrical field is capable of inducing the deformation of piezoelectric materials, which in turn provides mechanical stimulation for osteoblasts to aid bone healing [164]. Hence, the combination of EMFs and piezoelectric materials has been investigated. Among the piezoelectric materials, polyvinylidene fluoride (PVDF) is an attractive synthetic polymer in BTE owing to its excellent piezoelectricity and good biocompatibility [167]. Mirzaei et al. [168] fabricated a scaffold composed of PVDF and polyaniline (PANI)-a polymer with excellent electrical conductivity. Their results showed that both the PVDF scaffold and PVDF/PANI scaffold had improved cell attachment and osteogenic differentiation under EMFs exposure, while the combination of EMFs and PVDF/PANI scaffold had a more significant effect [168]. Similar results were obtained by Dong et al. [47], and they speculated that the mechanical stimulation resulting from the interplay between EMFs and PVDF-coated PCL-TCP scaffolds contributed to the well cellular response. The prominent effect of a combination of PVDF-based material and EMFs is inspiring and holds promises in the field of BTE.

#### Magnetic nanocomposites (MNC)

Magnetic nanocomposites (MNC) are smart materials typically composed of magnetic nanoparticles (MNPs) and the other component which is either organic or inorganic [169]. It is believed that the incorporation of MNPs endows magnetic composite scaffolds with magnetic properties and better mechanical properties, both of which improve cell spreading, adhesion, differentiation, and ability to respond to an external magnetic field [170, 171]. Combined with externally applied magnetic fields, MNC can interact with cells and modulate cell biological processes, thus MNC have been exploited for a large range of biomedical applications such as BTE [172]. Both static magnetic fields and EMFs can provide magnetic cues for MNC thus possessing great promise for assisting BTE [173]. Research works on applying static magnetic fields to combine with MNC attract a great amount of attention and obtain satisfactory results in this field [174–176]. There are also quite a few scientific reports on concomitantly applying EMFs with MNC for BTE applications. Huang et al. [177] identified that BMSCs seeded on porous scaffolds that are composed of Fe_2_O_3_ nanoparticles_,_ n-HAp and PLLA have higher ALP activity after PEMF stimulation. Correspondingly, Zeng et al. [178] further demonstrated that in all PEMF-stimulated groups, cells cultured on MNPs-dispersed HA scaffolds had higher ALP values than that on HA scaffolds. These two reports implied the interactions between externally applied EMFs and MNPs would lead to enhanced osteogenic differentiation. And the osteogenic effect may attribute to well cell behaviour under the presence of MNC scaffolds and the externally applied EMFs. For instance, by using scanning electron microscopy, Moradian et al. [43] observed that the presence of EMFs significantly promoted cell attachment on the MNC scaffolds. Similarly, Meshkini et al. [179] identified that the combination of magnetic nanofibres and EMFs significantly promoted MSCs adhere to material surface, and the number of adhered cells increases in an MNC content-dependent manner, which implies that it is the interaction of MNC and external EMFs affect biological behaviour of the loaded cell. Additionally, a combination of Mg.ATP-modified magnetic nanofibres and EMFs seems to be a great candidate in BTE applications [179]. The introduction of magnesium (Mg) and adenosine 5'-triphosphate (ATP), both of which are beneficial for osteogenic differentiation, allows the magnetic nanofibres to work synergistically with EMFs on enhancing osteogenic differentiation of MSCs. Given the solid foundation provided by these studies, it can be concluded that EMFs’ stimulation is, besides static magnetic fields, a potential tool to provide magnetic cues for MNC-based BTE.

**Fig. 5:**
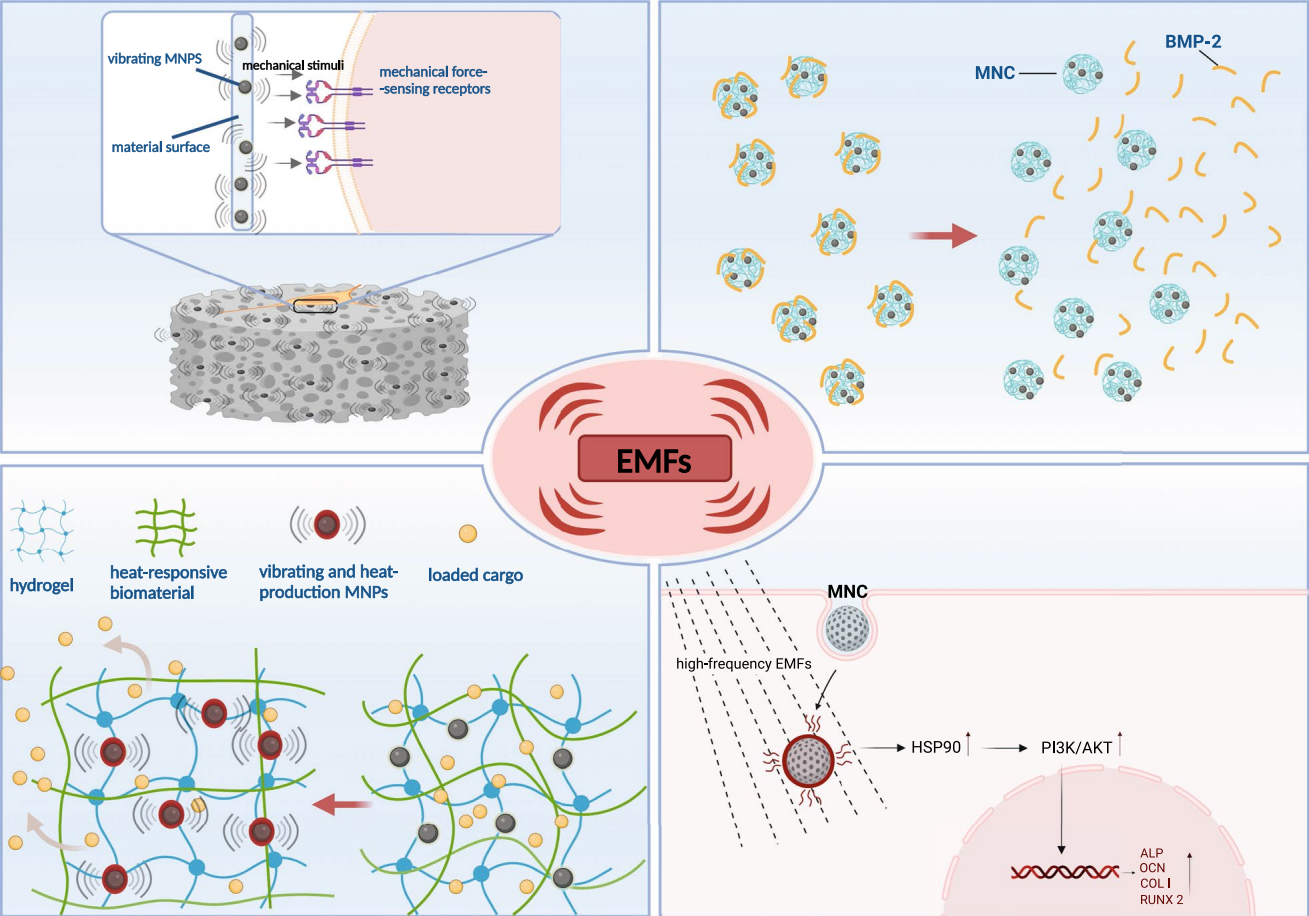
Interactions of MNC and cells under EMFs’ exposure. (1) Vibrations of MNPs induced by the presence of EMF provide mechanical cues for cells load on MNC [180]. (2, 3) Vibrations and generated heat of MNPs in the presence of EMFs affect the release of biological factors such as BMP-2 which would subsequently exert biological effects on adjacent cells [139, 181]. (4) Mild heat generated by MNC under high-frequency EMFs contributes to enhanced osteogenesis [186]. *MNC* magnetic nanocomposites, *EMFs* electromagnetic fields, *MNPs* magnetic nanoparticles, *BMP-2* bone morphogenetic protein-2, *HSP 90* heat shock protein 90. This image was drawn by the authors. Created with BioRender.com

The combination of EMFs and MNC exploits interactions between MNPs and the externally applied field. The interactions between MNC-based scaffold and loaded cell under magnetic force have been reviewed by Filippi et al. [172], whereas distinction from the constant magnetic force provided by static magnetic fields, EMFs such as PEMF, offers oscillating magnetic force. It is postulated that the interactions of MNC and loaded cells induced by EMFs include the following aspects. First, oscillating magnetic force can induce mechanical vibration of each MNP in the MNC scaffold [172]. The vibration of MNPs at the interface of MNC scaffold provides mechanical stimulation to cells, which might subsequently influence the ion channels, and activate the mechanotransduction pathway [180]. The vibration of MNPs induced by EMFs can also be exploited to control drug release. Thus, it is feasible to remotely control biological factor delivery and release by EMFs’ application. For instance, it has been identified that vibration resulting from EMFs is capable of promoting BMP-2 to release from MNC microspheres, even though the BMP-2 proteins have a chemical link with these magnetic microspheres [139]. In addition to vibration, heat generated by MNPs under EMFs exposure can also affect drug release. Zhang et al. [181] incorporated MNPs and thermo-responsive agar into polyethylene glycol (PEG) hydrogel, and the added agar was expected to change its network due to the generated heat of MNPs. Therefore, under the vibration and generated heat of MNPs, the release of drug was significantly promoted when EMFs were applied [181]. Accumulating studies indicated that mild heat generated by external heat sources could contribute to osteogenic differentiation and mineralization of MSCs by inducing upregulation of heat shock protein (HSP) and further activating the downstream signalling pathways [182–184]. Consequently, the thermal effect of MNPs under EMFs can be employed to facilitate osteogenic differentiation and lead to accelerated bone regeneration. Cao et al. [185] identified that MNPs-incorporated chitosan hydrogel generated mild heat under high-frequency EMFs, and the generated mild heat promoted osteogenic differentiation ability of MSCs in vitro. Wang et al. [186] further demonstrated that mild heat generated by MNPs under high-frequency EMFs facilitated osteogenesis by inducing HSP90 accumulation and the subsequent activation of PI3K/Akt pathway. The interactions of MNC and cells in the presence of EMFs are summarized in Fig. 5.

## EMFs promote the benefits of tissue-engineered scaffold on loaded cells

Bone-tissue-engineered scaffold, the most essential part of BTE, evolved from the initial biological substance that simply provides mechanical support to the current biomaterials that are tailored to possess osteoinductive or osteoconductive characteristics [5]. In most studies that employed EMFs to assist BTE, biological effects of EMFs on the loaded cell were mainly focused; in contrast, the benefits of tissue-engineered scaffold were rarely discussed. Undoubtedly, tissue-engineered scaffold essentially benefits the bone regeneration within bone defects under the circumstance of harnessing EMFs to assist BTE. Owing to the lack of direct evidence for clarifying the benefits of tissue-engineered scaffold, we can only indirectly understand the real role of tissue-engineered scaffold in bone defects treatment using the triad of stem cells, materials, and EMFs exposure by biological changes of cells on different scaffold. The presence of scaffolds dramatically affects biological effects induced by EMFs is firmly justified based on studies reviewed above. For instance, Schwartz et al. demonstrated that PEMF promoted osteogenesis by stimulating osteoprotegerin (OPG) production in osteoblasts. And they found that under the PEMF exposure, osteoblasts cultured on calcium phosphates showed significantly higher OPG production than the osteoblasts cultured on tissue culture polystyrene plastic [131]. Thus, it is believed that EMFs exposure involved in and promoted the positively biological effects supported by tissue-engineered scaffold on loaded cell. A recent study further indicated that the mechanical microenvironments established by tissue-engineered scaffold elicited distinct mechanotransduction responses and ultimately influenced MSCs responses to EMFs’ stimulation. In other words, EMFs influenced the signalling pathways activated by mechanical microenvironments of scaffolds, and further augmented the biological effects [187].

## Clinical applications of EMFs in bone defect treatment

A huge amount of in vitro studies builds a solid foundation for the clinical applications of EMFs in dealing with the skeletal disorder. Numerous clinical studies were carried on determining the clinical implications of PEMF, the FDA-approved electromagnetic field therapy. Ehnert et al. [37] summarized clinical studies on the effect of PEMF treatment on bone fracture non-unions, osteotomies, acute bone fractures, spinal fusion, osteoporosis, and osteoarthritis. And they concluded that EMFs presented a valuable adjunctive therapy for bone and osteochondral defects [37]. The clinical studies have indicated that PEMF presented a beneficial treatment in the management of delayed union and nonunion fractures, both of which are major complications in the treatment of skeletal defects [188]. For instance, in a prospective randomized controlled study that enrolled 58 patients, patients who received PEMF treatment for an average duration of 4.8 months had a union rate of 77.4%, whereas patients in the control group only had a successful union rate of 48.1% [189]. Spinal fusion surgery is usually performed when orthopaedic defects occur at load-bearing spine which is essential to the mobility of patients [5]. A randomized double-blind prospective study demonstrated that 92.2% of patients in PEMF treatment group had a successful fusion as determined by blinded radiographic evaluation, while the control group had a success rate of 67.9% [190]. Other clinical trials also advocated the application of PEMF for spinal fusion [191, 192]. Conclusively, PEMF proves its benefits in clinically treating bone defects. However, scant clinical data exist to demonstrate the efficiency and feasibility of a combination of EMFs and BTE in treating bone defects.

## Conclusion and future perspectives

Conclusively, the excellent performance of EMFs in bone regeneration renders it a promising adjunctive therapy for BTE. EMFs represents a non-invasive, and non-contact treatment, it has been exploited for many biomedical applications. For instance, PEMF, the most widely applied and FDA-approved electromagnetic field, has been put into clinical treatment of fractures, osteoarthritis, and osteoporosis for many years, whereas PEMF has not been clinically applied for bone defects that required bone substitutes. For critical-sized or large bone defects, simple EMFs’ stimulation can hardly help for bone regeneration within defects, whereas, when combined with stem cell-loaded scaffolds, EMFs show the surprising ability of facilitating bone regeneration. Consequently, EMFs that provide biophysical stimuli can be regarded as a promising complement to the classic triad of cells, materials, and biochemical factors in BTE [193, 194]. We summarized some advantages of EMFs in assisting BTE including cell adhesion enhancement, stem cells osteogenic differentiation augment, angiogenesis improvement, and M2 macrophage induction. Therefore, by remotely affecting multiple cell types and their biological behaviour, EMFs seem perfectly suited for BTE applications. Additionally, the employment of EMFs does help for addressing some hurdles in BTE applications, such as the suboptimal osteointegration of hydroxyapatite and Ti implant. However, there still exist some limitations in EMFs’ application. As is known, the biological effects induced by EMFs may vary with many parameters including frequency, continuous exposure time, and even directionalities [195]. It implies that much work remains to be done before determining the best parameters capable of inducing the desired biological effects. Meanwhile, a poor understanding of the underlying mechanisms further damps the broad clinical application of EMFs [37]. To build a solid foundation for EMFs’ applications in BTE, future studies are required to clarify the safety, optimal EMFs parameter settings for human exposures as well as the molecular and cellular mechanisms of EMFs on the living organism.

EMFs have shown promises in combining diverse material types and their combinations, especially materials with piezoelectricity and materials with magnetic responsiveness. Piezoelectric materials under external electric fields generate structural deformation, which subsequently leads to the generation of electric activity. It is the actuated deformation and electric activity contribute to the strengthened performance of piezoelectric materials under EMFs exposure. Moreover, MNC and MNPs are attractive materials owing to their exceptional properties and excellent performance in biomedical applications. The vibration and induced heat of MNPs under EMFs exposure confer MNC with the capability of stimulating osteogenesis and remote controlling of biological factors release. These materials that can respond to external stimuli are called stimuli-responsive biomaterials. Stimuli-responsive biomaterials bring a great deal of attention to the field of BTE as they can partially recapitulate the dynamic environment of living tissue under external stimuli [196]. Externally applied EMFs emerging as a potential external stimulus. This is because EMFs can not only directly influence cells, but also interact with bone tissue engineering materials and subsequently promote osteogenesis. The combination of EMFs and stimuli-responsive biomaterials is promising in BTE application. Further studies are needed for deepening our knowledge of interactions between EMFs and BTE scaffolds, which in turn would help for better designing of scaffolds aimed at EMFs-assisted BTE. In summary, EMFs as an ideal adjunctive therapy hold great potential in combining with diverse biomaterials for BTE applications. That harnessing EMFs to assist successful performance of biomaterials would be an effective strategy for bone defects treatment.

## Data Availability

Data sharing is not applicable to this article as no new data were created or analysed in this study.

## Abbreviations

BTE: Bone tissue engineering
EMFs: Electromagnetic fields
MCs: Mesenchymal stem cells
PEMF: Pulsed electromagnetic fields
SEMF: Sinusoidal electromagnetic field
RGD: Arginine-glycine-aspartic acid
ECM: Extracellular matrix
ALP: Alkaline phosphatase
Runx2: Runt-related transcription factor 2
VEGF: Vascular endothelial growth factor
FGF: Fibroblast growth factor
PCL: Poly(ε-caprolactone)
BMSCs: Bone marrow-derived mesenchymal stem cells
iPSCs: Induced pluripotent stem cells
ADSCs: Adipose-derived mesenchymal stem cells
PLGA: Poly(lactic-co-glycolic acid)
HAp: Hydroxyapatite
Ti: Titanium
BMP-2: Bone morphogenetic protein-2
OCN: Osteocalcin
Col-1: Collagen I
OSX: Osterix
PLLA: Poly(l-lactic acid)
CMC: Carboxymethyl chitosan
PVDF: Polyvinylidene fluoride
PANI: Polyaniline
MNC: Magnetic nanocomposites
MNPs: Magnetic nanoparticles
